# Meal phosphate variability does not support fixed dose phosphate binder schedules for patients treated with peritoneal dialysis: a prospective cohort study

**DOI:** 10.1186/s12882-015-0205-3

**Published:** 2015-12-09

**Authors:** Simon Leung, Brendan McCormick, Jessica Wagner, Mohan Biyani, Susan Lavoie, Rameez Imtiaz, Deborah Zimmerman

**Affiliations:** Division of Endocrinology, Department of Medicine, Ottawa Hospital, Ottawa, ON Canada; Division of Nephrology, Department of Medicine, Ottawa Hospital, University of Ottawa, Ottawa, ON Canada; Kidney Research Centre, Ottawa Hospital Research Institute, Ottawa, ON Canada; Department of Medicine, University of Ottawa, Ottawa, ON Canada; Riverside Campus of the Ottawa Hospital, 1967 Riverside Dr, Ottawa, ON K0A 2Z0 Canada

**Keywords:** Peritoneal dialysis, Phosphate, Diet, Variability

## Abstract

**Background:**

Removal of phosphate by peritoneal dialysis is insufficient to maintain normal serum phosphate levels such that most patients must take phosphate binders with their meals. However, phosphate ‘counting’ is complicated and many patients are simply prescribed a specific dose of phosphate binders with each meal. Therefore, our primary objective was to assess the variability in meal phosphate content to determine the appropriateness of this approach.

**Methods:**

In this prospective cohort study, adult patients with ESRD treated with peritoneal dialysis and prescribed phosphate binder therapy were eligible to participate. Participants were excluded from the study if they were unable to give consent, had hypercalcemia, were visually or hearing impaired or were expected to receive a renal transplant during the time of the study. After providing informed consent, patients kept a 3-day diet diary that included all foods and beverages consumed in addition to portion sizes. At the same time, patients documented the amount of phosphate binders taken with each meal. The phosphate content of the each meal was estimated using ESHA Food Processor SQL Software by a registered dietitian. Meal phosphate and binder variability were estimated by the Intra Class Correlation Coefficient (ICC) where 0 indicates maximal variability and 1 indicates no variability.

**Results:**

Seventy-eight patients consented to participate in the study; 18 did not complete the study protocol. The patients were 60 (±17) years, predominately male (38/60) and Caucasian (51/60). Diabetic nephropathy was the most common cause of end stage kidney disease. The daily phosphate intake including snacks ranged from 959 ± 249 to 1144 ± 362 mg. The phosphate ICC by meal: breakfast 0.63, lunch 0.16; supper 0.27. The phosphate binder ICC by meal: breakfast 0.68, lunch 0.73, supper 0.67.

**Conclusion:**

The standard prescription of a set number of phosphate binders with each meal is not supported by the data; patients do not appear to be adjusting their binders to match the meal phosphate content. An easy to use phosphate counting program that assists the patient in determining the appropriate amount of phosphate binder to take may enhance phosphate control.

## Background

Helping patients with end stage renal disease (ESRD) maintain a normal serum phosphate level remains a challenge for many healthcare practitioners. Hyperphosphatemia, a condition in which the renal filtration and excretion of phosphate decreases as chronic kidney disease (CKD) advances, has been independently associated with adverse outcomes including abnormal bone and mineral metabolism [1], vascular and soft tissue calcification [2, 3], and cardiovascular morbidity and mortality in patients treated with hemodialysis and peritoneal dialysis [4–6]. In an attempt to mitigate this risk, the 2009 Kidney Disease Improving Global Outcomes (KDIGO) guidelines included targets for serum phosphate [7], but several studies have shown that achieving serum phosphate of 3.5 and 5.5 mg/dL (1.1–1.8 mmol/L) is difficult for a large portion of patients treated with dialysis [5, 8–10].

The treatment of hyperphosphatemia in dialysis patients includes many factors: adequate dialysis, dietary phosphate restriction, and phosphate-binding agents. Conventional hemodialysis and peritoneal dialysis provide inadequate phosphate removal alone such that almost all patients will be in a positive phosphate balance [11]. Nutritional interventions to control phosphate intake attempt to limit the amount of daily phosphate from foods to 800 to 1100 mg of phosphate per day [7]. In addition to dietary prescriptions, intense educational strategies to increase patient knowledge about dietary phosphate have been shown to be effective in lowering serum phosphate, but long interventions (≥4 months) may be needed for sustained benefit [12, 13]. Dietician efforts are hampered by poor compliance to diet which is a prevalent theme among patients treated with dialysis [14–17]. Also, overly restrictive diets may be complicated to follow and may negatively impact a patient’s nutritional status, resulting in protein calorie malnutrition and a greater risk of death [18]. Lastly phosphate binders can be taken with meals to limit intestinal absorption of phosphate; none are without their limitations. Since its introduction, calcium carbonate remains in wide use today primarily because of its low cost compared to other phosphate binders. Calcium carbonate, however, may predispose some dialysis patients to hypercalcemia, especially if the dosing is mistimed with meals [11]. Despite the presumed variability in daily meal phosphate consumption, it is common to prescribe a fixed dose of phosphate binders that does not necessarily mirror the day-to-day and meal-to-meal changes in phosphate intake. This variation in phosphate intake could lead to a mismatch between phosphate intake and phosphate binder dose resulting in an increase in phosphate or calcium absorption [19]. Unnecessarily increasing phosphate binder fixed dosing in an attempt to reduce serum phosphate can increase pill burden and contribute to reduced medication adherence [20].

Based on the premise that self-adjustment of phosphate binders by dialysis patients using a mobile application may improve phosphate control, the aim of this 3-phase study is to develop a novel phosphate counting program for the Apple iPod touch device. Phase 1 of the study was to determine the daily meal phosphate variability to ensure that development of such an application is warranted.

## Methods

The study was approved by the Ottawa Health Science Research Ethics Network (ID 20120105-01H). Patients were recruited from The Ottawa Hospital Home Dialysis Unit in Ottawa, Canada from July 2012 through October 2014. English or French speaking/writing adult patients who had ESRD treated with peritoneal dialysis and phosphate binder therapy were eligible for participation. Participants were excluded from the study if they (i) were unable or unwilling to give informed consent, (ii) had hypercalcemia, (iii) were visually or hearing impaired or (iv) were expected to receive a renal transplant during the time of the study. After obtaining informed consent, patients were taught to use a registered dietitian (RD) developed food journal. One week prior to their next routine clinic visit, participants were asked to document their food and beverage intake, and the number of phosphate binders taken at meals for 3 days using the food journals. Pre-clinic biochemistry tests including serum calcium, phosphate, and parathyroid hormone, and a standard adequacy assessment using the Baxter Adequest program (Baxter Healthcare, Deerfield, Il) were performed. The completed food journals were reviewed by the RD to ensure accuracy; patients were contacted as necessary to confirm serving sizes, brand names, and food type. To calculate the phosphate content of the foods and beverages, the ESHA Food Processor SQL Software was used; although infrequent, other sources of data were used for foods not found in the software (e.g. certain cheeses, cereals, and snacks) including published manuscripts (USDA National Nutrient Database for Standard Reference, Release 25), government nutrient databases (Food Standards Australia New Zealand’s NUTTAB 2010 Online Searchable Database), and the food manufacturer.

Data including basic patient characteristics was entered into an Excel spreadsheet. Summary descriptive statistics to describe the population, mean and standard deviation for phosphate intake and phosphate binder dose taken were calculated. Box and whisker plots for the 3-day phosphate intake by meal were created. Intraclass correlation coefficients (ICC) for meal phosphate content and phosphate binders taken with the meals were calculated using SAS 9.3 SAS Institute Inc., Cary, NC, USA. The ICC is a descriptive statistic that allows an assessment of how strongly units (e.g. phosphate) in the same group (e.g. breakfast) are similar to each other. The ICC value ranges from 0 to 1; as the ICC increases the more the units in the group resemble each other. An ICC of > 0.75 suggests that the units in the group are very similar to each other.

## Results

### Participants and recruitment

A total of 150 patients were screened for the study. Twenty-two patients were not eligible (prior to consent 3 patients died, 9 patients received renal transplants, 6 patients switched to hemodialysis, 2 patients recovered renal function, and 2 patients were not taking a phosphate binder). Fifty patients declined to participate. Seventy-eight peritoneal dialysis patients consented to the study, 60 (38 men and 22 women) completed the study protocol (Table 1). Reasons for participant withdrawal from the study included: 3 received a kidney transplant, 1 died, 5 withdrew consent, 7 were unable or unwilling to follow the protocol, and 2 were found to be ineligible.

**Table 1:** Baseline patient demographics

Patient Characteristics	
Age – Years (SD)	60 (17)
Sex (M:F)	38:22
Race N (%)	Caucasian 51 (85) Aboriginal 1 (2) African American 3 (5) Asian 5 (8)
DM N (%)	30 (50)
Etiology of ESRD N (%)	DM 18 (30) PKD 8 (13) GN 6 (10) Other 28 (47)
Calcium Based Binders N (%)	57 (95)
Sevelemer N (%)	6 (10)

On average patients were 60 ± 17 years old, predominately Caucasian and 50 % had a history of diabetes mellitus (Table 1). The majority of patients were taking calcium carbonate as their phosphate binder with an average prescribed elemental calcium dose per day of 800 ± 440 mg per day. Only 6 of the patients were using sevelamer hydrochloride as a phosphate binder. Total daily phosphate intake including snacks over the 3 days ranged from 959 ± 249 mg to 1144 ± 362 mg per day with the majority of phosphate being consumed with supper (Table 2).

**Table 2:** Meal phosphate content and phosphate binder dose by meal

^a^Daily mean phosphate intake in mg (SD)	Phosphate binder intake (mg)	Meal	Mean meal phosphate intake in mg (SD)
1144 (362)	627 (542)	Breakfast (Day 1)	215 (162)
	848 (727)	Lunch	302 (247)
	978 (684)	Supper	566 (186)
973 (283)	581 (589)	Breakfast (Day 2)	183 (83)
	678 (726)	Lunch	256 (156)
	959 (732)	Supper	462 (218)
959 (249)	545 (558)	Breakfast (Day 3)	239 (119)
	686 (713)	Lunch	233 (148)
	1000 (726)	Supper	437 (158)

^a^total includes snacks

*mg* milligrams, *SD* standard deviation

**Fig. 1:**
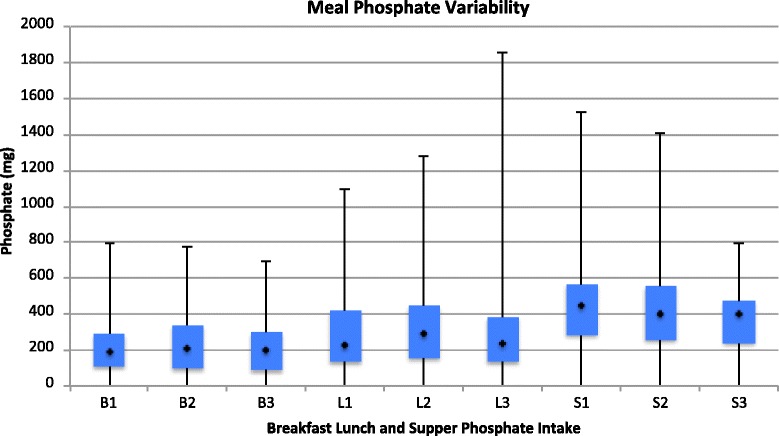
Meal Phosphate Variability

Meal phosphate intake ranged from a minimum to a maximum of 0 to 798 mg for breakfast, 0 to 1853 mg for lunch and 0 to 1523 mg for supper (Fig. 1). There was also tremendous variability in the amount of phosphate consumed with snacks ranging from 0 to 463 mg per snack with some patients consuming 3 snacks in 1 day. The ICCs meal phosphate content was: Breakfast = 0.63; Lunch = 0.16; Supper = 0.27. The ICCs for phosphate binders taken with the meals were: Breakfast = 0.68; Lunch = 0.73; Supper = 0.67.

## Discussion

In our prospective cohort of patients with ESRD treated with peritoneal dialysis, we have shown that the ICCs associated with meal phosphate content, with the exception of breakfast, are highly variable and do not support a standard number of phosphate binders to be order for each meal. Despite dietician review at each clinic visit, the ICCs for meal binder use suggest that patients follow fixed binder dosing as prescribed and are not varying their phosphate binders to the extent that meal phosphate content changes. This variability in meal phosphate intake with relatively fixed phosphate binder dosing may contribute to the poor phosphate control experienced by patients on dialysis. Our results are in agreement with one previous study of 16 pediatric patients with chronic kidney disease [21].

In spite of these issues, normalizing serum phosphate levels with phosphate binders is possible. During the first 12 weeks in the Treat-to-Goal study involving 200 hemodialysis patients, the phosphate binder dose was titrated every 3 weeks until the patient’s serum phosphate concentration fell between 3.0 to 5.0 mg/dL (1–1.6 mmol/L) [22]. However, it is possible that mismatched timing of meal phosphate intake and binder dose may lead to an increased number of phosphate binders being taken than are required. Better knowledge of phosphate dietary restrictions may improve serum phosphate [23]; Karavetian et al. found in a recent meta-analysis that all but one of the 18 studies that met their inclusion criteria reported either a significant (14 of the 18 studies) or a non-significant reduction in serum phosphate levels, which ranged from 0.3 to 1.6 mg/dL (average: 1 mg/dL; 0.01–0.5 mmol/L), when hemodialysis patients received nutrition counseling. The interventions were highly variable and lasted 20–40 minutes in just one session to as many as eight sessions per month for 1 to 6 months [13]. The authors identified the effective strategies that contributed to dietary behavior change included high-intensity education, long duration of intervention, and individualized counseling by a renal dietitian. For example in a study by Yokum et al., serum phosphate levels were significantly reduced in the intervention group compared to standard of care group over a 4 month period but both a dietitian and pharmacist were part of the phosphate lowering intervention strategy [24]. Although this intensive and time consuming education appears to be effective in reducing serum phosphate, time may be a limited commodity for dietitians in most dialysis centers such that achieving phosphate control may be very difficult in a routine clinical care setting [25].

To further complicate the issue, more education time does not necessarily equate to improvements in serum phosphate concentration over time. Ashurst et al. provided participants in their intervention group with a onetime 40-min dietetic education session just prior to hemodialysis, and this intervention was shown to significantly reduce and maintain the serum phosphate levels over a 3 month period [26]. However in a study by Morey et al. study, monthly dietetic consultation resulted in significantly decreased serum phosphate levels by the third month, but by the six month these levels were neither sustained nor significantly different from baseline [27]. Importantly, although the study protocol and the length of the intervention periods of Yokum et al., Morey et al., and Ashurst et al. differed, all three studies also included education regarding the proper use of phosphate binders. None of the authors distinguished how much of the improvements in serum phosphate levels was a result of dietary restriction versus phosphate binder adherence. Thus, a simpler approach that combines both dietary and phosphate binder educational elements into one novel tool to help dialysis patients manage serum phosphate levels may be useful.

An innovative concept was developed to account for the discrepancy between the phosphate binder dose and the differences in phosphate quantities at meals. Patients who were instructed in the ‘Phosphate Education Program’ (PEP) would self-adjust his or her phosphate binder dose to the amount of phosphate at each meal by first eye-estimating the amount of phosphate using ‘Phosphate Units’ (PU). PU was a predefined amount of phosphate in a food based on the major food group it belongs to, and then the patient would apply a phosphate binder/PU ratio prescribed by the physician titrated to the patient’s predialysis serum phosphate concentration [19, 21]. This concept was trialed with pediatric CKD patients in a non-randomized prospective study (*n* = 16), which found that self-adjustment of phosphate binder dose in relation to meal phosphate content improved the management of hyperphosphatemia within 6 weeks after PEP training but the statistically significant effect was not sustained. PEP training included a workshop, an individualized consult with the dietitian, and a separate meeting with the pediatric nephrologist [21]. The PEP improves upon the current practice of fixed phosphate binder doses, but this approach may be too difficult for many patients.

Mobile applications can be practical tools in the self-management of diabetes and are preferred for their ease of use over web- or computer-based programs [28]. Importantly, mobile applications have been shown to improve glycemic control in people living with type-1 diabetes [29, 30]. Although several technology-based tools for the management of CKD are available, a novel ‘phosphate counting’ program for the Apple iPod touch device to teach patients how to ‘match’ phosphate binders to the amount of phosphate in food does not exist. Development of such an APP may increase phosphate binder adherence, better match phosphate binder dose to phosphate intake, and improve serum phosphate levels.

## Conclusion

The standard practice of prescribing fixed doses of phosphate binders at meals is an inappropriate management strategy for the control of serum phosphate concentration as meal phosphate intake is highly variable as was demonstrated in this study. Intensive dietary education remains a cornerstone of phosphate management, but such interventions are time consuming and dialysis facilities may not have sufficient resources to meet the educational needs of their patients. Although an innovative approach to empower patients to self-adjust their phosphate binder dose based on phosphate intake has been developed and tested, a simpler method that takes advantage of mobile application technology may improve phosphate binder compliance and management of hyperphosphatemia.
